# Generation of enterocyte-like cells from human induced pluripotent stem cells for drug absorption and metabolism studies in human small intestine

**DOI:** 10.1038/srep16479

**Published:** 2015-11-12

**Authors:** Tatsuya Ozawa, Kazuo Takayama, Ryota Okamoto, Ryosuke Negoro, Fuminori Sakurai, Masashi Tachibana, Kenji Kawabata, Hiroyuki Mizuguchi

**Affiliations:** 1 Laboratory of Biochemistry and Molecular Biology, Graduate School of Pharmaceutical Sciences, Osaka University, Osaka 565-0871, Japan; 2 Laboratory of Hepatocyte Regulation, National Institute of Biomedical Innovation, Health and Nutrition, Osaka 567-0085, Japan; 3 iPS Cell-based Research Project on Hepatic Toxicity and Metabolism, Graduate School of Pharmaceutical Sciences, Osaka University, Osaka 565-0871, Japan; 4 Laboratory of Regulatory Sciences for Oligonucleotide Therapeutics, Clinical Drug Development Project, Graduate School of Pharmaceutical Sciences, Osaka University Osaka 565-0871, Japan; 5 Laboratory of Stem Cell Regulation, National Institute of Biomedical Innovation, Health and Nutrition, Osaka 567-0085, Japan; 6 Laboratory of Biomedical Innovation, Graduate School of Pharmaceutical Sciences, Osaka University, Osaka 565-0871, Japan; 7 Global Center for Medical Engineering and Informatics, Osaka University, Osaka 565-0871, Japan

## Abstract

Enterocytes play an important role in drug absorption and metabolism. However, a widely used enterocyte model, Caco-2 cell, has difficulty in evaluating both drug absorption and metabolism because the expression levels of some drug absorption and metabolism-related genes in these cells differ largely from those of human enterocytes. Therefore, we decided to generate the enterocyte-like cells from human induced pluripotent stem (iPS) cells (hiPS-ELCs), which are applicable to drug absorption and metabolism studies. The efficiency of enterocyte differentiation from human iPS cells was significantly improved by using EGF, SB431542, and Wnt3A, and extending the differentiation period. The gene expression levels of *cytochrome P450 3A4* (CYP3A4) and *peptide transporter 1* in the hiPS-ELCs were higher than those in Caco-2 cells. In addition, CYP3A4 expression in the hiPS-ELCs was induced by treatment with 1, 25-dihydroxyvitamin D3 or rifampicin, which are known to induce CYP3A4 expression, indicating that the hiPS-ELCs have CYP3A4 induction potency. Moreover, the transendothelial electrical resistance (TEER) value of the hiPS-ELC monolayer was approximately 240 Ω*cm^2^, suggesting that the hiPS-ELC monolayer could form a barrier. In conclusion, we succeeded in establishing an enterocyte model from human iPS cells which have potential to be applied for drug absorption and metabolism studies.

It is known that poor pharmacokinetics and poor bioavailability are responsible for approximately 10% of drug withdrawal^1^. Various organs, including the small intestine, play an important role in pharmacokinetics (absorption, distribution, metabolism, and excretion) and bioavailability. Because the drug transporters and metabolism enzymes are strongly expressed in enterocytes, which are the primary site of drug absorption after oral administration, the small intestine plays a major role in drug absorption and metabolism^2^. Specifically, cytochrome P450 3A4 (CYP3A4, the dominant drug metabolizing enzyme in the human small intestine), peptide transporter 1 (PEPT1), and P-glycoprotein (P-gp) are expressed at high levels in the enterocytes^3^,^4^. Because CYP3A4 interacts with absorbed drugs in the enterocytes, CYP3A4-mediated intestinal metabolism is a significant factor in oral drug bioavailability^5^,^6^. In addition, CYP3A4 and P-gp share not only many substrates (e.g., dexamethasone and etoposide) and inhibitors (e.g., quinidine and testosterone), but also inducers such as rifampicin^7^. Therefore, a model that could evaluate both drug absorption and metabolism would greatly facilitate the development of safer and more effective drugs.

Animal models are used for evaluation of drug absorption in the small intestine. However, it is known that there are species differences in small intestinal drug absorption and the first-pass effect. Because human primary enterocytes cannot be obtained in large numbers, Caco-2 cells (a human colorectal carcinoma cell line) monolayers are widely utilized for drug absorption studies in human small intestine^8^,^9^,^10^. Polarized Caco-2 cell monolayers can form a physical and biochemical barrier that reproduces the enterocyte barrier of the human small intestine. Although the Caco-2 cell monolayer is a useful model, it does have some significant drawbacks. First, it is difficult to accurately evaluate CYP3A4-mediated drug metabolism and the CYP3A4 induction potency of drugs because the CYP3A4 expression levels in Caco-2 cells are much lower than those in the enterocytes^11^,^12^. Consequently, it is difficult to evaluate both drug absorption and metabolism by using the Caco-2 cell monolayer model. Secondly, the permeability level of hydrophilic drugs, which are absorbed via the paracellular route, in Caco-2 cells is lower than that in the human small intestine^10^,^13^. Therefore, it is also difficult to evaluate the hydrophilic drug absorption by using the Caco-2 cell monolayer model. There is thus, need of a novel model to resolve these issues.

Human induced pluripotent stem (iPS) cells^14^ have the potential to self-replicate and differentiate into multiple types of body cells, including enterocytes. In this study, we aimed to generate enterocyte-like cells from human iPS cells (hiPS-ELCs) which could evaluate both drug absorption and metabolism. Recently, some groups have reported that intestinal tissues and intestinal organoids, which are consist of all four intestinal cell types (paneth cells, goblet cells, enterocytes, and enteroendocrine cells), could be differentiated from human pluripotent stem cells *in vitro*^15^,^16^,^17^,^18^. However, it might be difficult to apply the intestinal tissues to drug absorption and metabolism studies because they are generated under 3-dimensional culture conditions. Although some researchers have demonstrated that enterocyte-like cells could be generated under 2-dimensional culture conditions, their drug absorption and metabolism capacities have not been sufficiently characterized^19^,^20^,^21^. Therefore, we decided to generate enterocyte monolayers, which have drug absorption and metabolism capacities, from human iPS cells.

## Materials and Methods

### Reagents for compound screening

PMA was purchased from BIAFFIN GMBH & CO KG. TGFβ1, EGF, Follistatin, and R-spondin 1 were purchased from R&D systems. Wortmannin and Pentagastrin were purchased from Sigma. Noggin was purchased from PeproTech. SB431542 was obtained from Wako. To generate Wnt3A-conditional medium, L-Wnt-3A cells (ATCC, CRL2647) were cultured with the DMEM-High Glucose medium (Invitrogen), which contains 10% Knockout Serum Replacement, 1% Non-Essential Amino Acid Solution, Penicillin-Streptomycin, 2 mM L-Glutamine, and 100 μM β-mercaptoethanol, for 24 hr, and then the conditioned medium was collected.

### Human iPS cells culture

A human iPS cell line, Tic^22^,^23^ (provided by Dr. A. Umezawa, National Center for Child Health and Development), was maintained on a feeder layer of mitomycin C-treated mouse embryonic fibroblasts (Millipore) with ReproStem medium (ReproCELL) supplemented with 10 ng/ml fibroblast growth factor 2 (FGF2, KATAYAMA CHEMICAL INDUSTRIES).

### *In vitro* differentiation

Before the initiation of enterocyte differentiation, human iPS cells were dissociated into clumps by using dispase (Roche) and plated onto BD Matrigel Basement Membrane Matrix (BD Biosciences). These cells were cultured in the MEF-conditioned medium for 2–3 days. The differentiation protocol for the induction of definitive endoderm cells was described previously^24^. Briefly, for the definitive endoderm differentiation, human iPS cells were cultured for 4 days in L-Wnt3A-expressing cell-conditioned RPMI1640 medium (Sigma) containing 100 ng/ml Activin A (R&D Systems), 4 mM L-Glutamine, 0.2~0.5% FBS, and 1 × B27 Supplement Minus Vitamin A (Life Technologies). For the induction of intestine-like cells, the definitive endoderm cells were cultured for 15 days in DMEM-High Glucose medium (Invitrogen) containing 5 μM 6-Bromoindirubin-3′-oxime (BIO; Calbiochem), 10 μM N-[(3,5-difluorophenyl) acetyl]-L-alanyl-2-phenyl-1, 1-dimethylethyl ester-glycine (DAPT; Peptide Institute), 10% Knockout Serum Replacement (Invitrogen), 1% Non-Essential Amino Acid Solution (Invitrogen), Penicillin-Streptomycin, 2 mM L-Glutamine, and 100 μM β-mercaptoethanol. For the induction of hiPS-ELCs, the intestine-like cells were cultured for 15 days in L-Wnt3A-expressing cell-conditioned DMEM-High Glucose medium (Invitrogen) containing 5 μM BIO, 10 μM DAPT, 10 μM SB431542 (Wako), 250 ng/ml EGF (R&D systems), 10% Knockout Serum Replacement, 1% Non-Essential Amino Acid Solution, Penicillin-Streptomycin, 2 mM L-Glutamine, and 100 μM β-mercaptoethanol.

### Caco-2 cells culture and differentiation

Caco-2 cells were cultured with DMEM-High Glucose medium (Invitrogen) containing 1 × HEPES (Invitrogen), 10% FBS, 1% Non-Essential Amino Acid Solution (Invitrogen), Penicillin-Streptomycin, and 4 mM L-Gln. For differentiation of Caco-2 cells, Caco-2 cells were cultured for 21 days after they reached confluence.

### RNA Isolation and Reverse Transcription-Polymerase Chain Reaction (RT-PCR)

Total RNA was isolated from Caco-2 cells, human iPS cells and their derivatives using ISOGENE (NIPPON GENE). cDNA was synthesized using 500 ng of total RNA (including Human Adult Intestine Total RNA (Clontech, BioChain: 4 lots)) with a Superscript VILO cDNA synthesis kit (Invitrogen). Real-time RT-PCR was performed with SYBR Green PCR Master Mix (Applied Biosystems) using a StepOnePlus real-time PCR system (Applied Biosystems). Relative quantification was performed against a standard curve and the values were normalized against the input determined for the housekeeping gene, *glyceraldehyde 3-phosphate dehydrogenase* (*GAPDH*). PCR primers sequences were described in Table. S1.

### Immunohistochemistry

To perform the immunohistochemistry, the human iPS-derived cells were fixed with 4% PFA in PBS for 10 min. After blocking the cells with PBS containing 10% FBS, 1% bovine serum albumin (BSA), and 1% Triton X-100 for 45 min, the cells were incubated with a primary antibody (described in Table S1) at 4 °C overnight, and finally, incubated with a secondary antibody (described in Table S1) at room temperature for 1 hr.

### Flow cytometry

Single-cell suspensions of the human iPS-derived cells were treated with 1 × Permeabilization Buffer (e-Bioscience), and then incubated with the primary antibody (anti-VILLIN antibody, (Abcam, ab109516)), followed by the secondary antibody (Goat Anti-Rabbit IgG APC-conjugated Antibody (R&D systems) or Donkey anti-Rabbit IgG (H + L) Secondary Antibody, Alexa Fluor 488 conjugate (Molecular probes)). Single-cell suspensions of the human iPS-derived cells were also incubated with the Ki-67 Antibody (Ki-67) FITC or Annexin V (FL) FITC (both from Santa Cruz Biotechnology). Flow cytometry analysis was performed using a FACS LSR Fortessa flow cytometer (BD Biosciences).

### Western Blotting

The human iPS-derived cells and Caco-2 cells were homogenized with lysis buffer (20 mM HEPES, 2 mM EDTA, 10% glycerol, and 1% Triton X-100) containing a protease inhibitor mixture (Sigma). After being frozen and thawed, the homogenates were centrifuged at 15,000 *g* at 4 °C for 15 min, and the supernatants were collected. The lysates were subjected to SDS-PAGE on 7.5% polyacrylamide gel, and then transferred onto polyvinylidene fluoride membranes (Millipore). After the reaction was blocked with 1% skim milk in TBS containing 0.1% Tween 20 at room temperature for 1 hr, the membranes were incubated with anti-human CYP3A4 or β-actin antibodies at 4 °C overnight, followed by reaction with horseradish peroxidaseconjugated anti-goat IgG antibodies or anti-mouse IgG antibodies, respectively, at room temperature for 1 hr. The band was visualized by Chemi-Lumi One Super (Nacalai Tesque) and the signals were read using an LAS-4000 imaging system (GE Healthcare).

### CYP3A4 activity

To measure the CYP3A4 activity, we performed lytic assays by using a P450-Glo CYP3A4 (catalog number; V9001) Assay Kit (Promega). We measured the fluorescence activity with a luminometer (Lumat LB 9507; Berthold) according to the manufacturer’s instructions. The CYP3A4 activity was normalized with the protein content per well.

### CYP3A4 and P-gp induction

The human iPS-derived cells were treated with 1,25-Dihydroxyvitamin D3 (Sigma)^10^ or rifampicin (Wako)^25^, which are known to induce CYP 3A4 and P-gp, at a final concentration of 100 nM for 24 hr or 20 μM for 48 hr. Controls were treated with DMSO (final concentration 0.1%).

### TEER measurements

TEER of human iPS-derived cells and Caco-2 cells, which were cultured on cell culture inserts (BD Biosciences) from day 0 of differentiation, was measured by Millicell-ERS (Merck Millipore). The raw data were converted to Ω × cm^2^ based on the culture insert area.

### Fluorescein isothiocyanate–dextran 4000 (FD-4) permeability tests

Human iPS-derived cells, Caco-2 cells, and undifferentiated human iPS cells, which were cultured on the cell culture inserts were washed Hank’s Balanced Salt Solution (HBSS; Life Technologies). HBSS containing 12.5 mg/ml FD-4 (Sigma) was added to the apical side, and HBSS was added to the basolateral side. After 90 min incubation at 37 °C, the solution was collected from the basolateral side. The concentration of FD-4 in the solution was measured by using the plate reader Sunrise (Tecan).

### Uptake of D-Ala-Leu-Lys-7-amido-4-methylcoumarin (AMCA)

To examine PEPT1 activity, the cells were treated with 25 μM D-Ala-Leu-Lys-AMCA (a fluorescence-labeled tripeptide which is a substrate of PEPT1; sigma) for 4 hr. To inhibit the PEPT1 activity level, the cells were pretreated with 100 μM captopril (sigma) for 24 hr before D-Ala-Leu-Lys-AMCA treatment.

### Cell viability

Cell viability was assessed by using a WST-8 assay kit (Dojindo). The cell viability of hiPS-ELCs (on day 19) was taken as 100.

## Results

### Improvement of the enterocyte differentiation efficiency by treatment with compounds and extension the differentiation period

Although many groups have demonstrated that intestinal cells could be differentiated from human pluripotent stem cells^16^,^19^,^20^,^21^,^26^, a protocol for enterocyte monolayer differentiation for drug absorption and metabolism studies has not been well established, to our knowledge. Ogaki *et al.* reported that the combination of BIO and DAPT treatments could promote intestinal differentiation from the definitive endoderm cells^26^. We also tried to perform intestinal differentiation by using BIO and DAPT treatment according to the protocol described in Fig. S1A. To examine the efficiency of enterocyte differentiation, the percentage of VILLIN-positive cells was measured by FACS analysis. The percentage of VILLIN-positive cells in the human iPS-derived intestinal cells was approximately 17% (Fig. S1B). Thus, it was necessary to improve the enterocyte differentiation efficiency in order to apply the hiPS-ELCs to drug pharmacokinetic studies.

**Figure 1:**
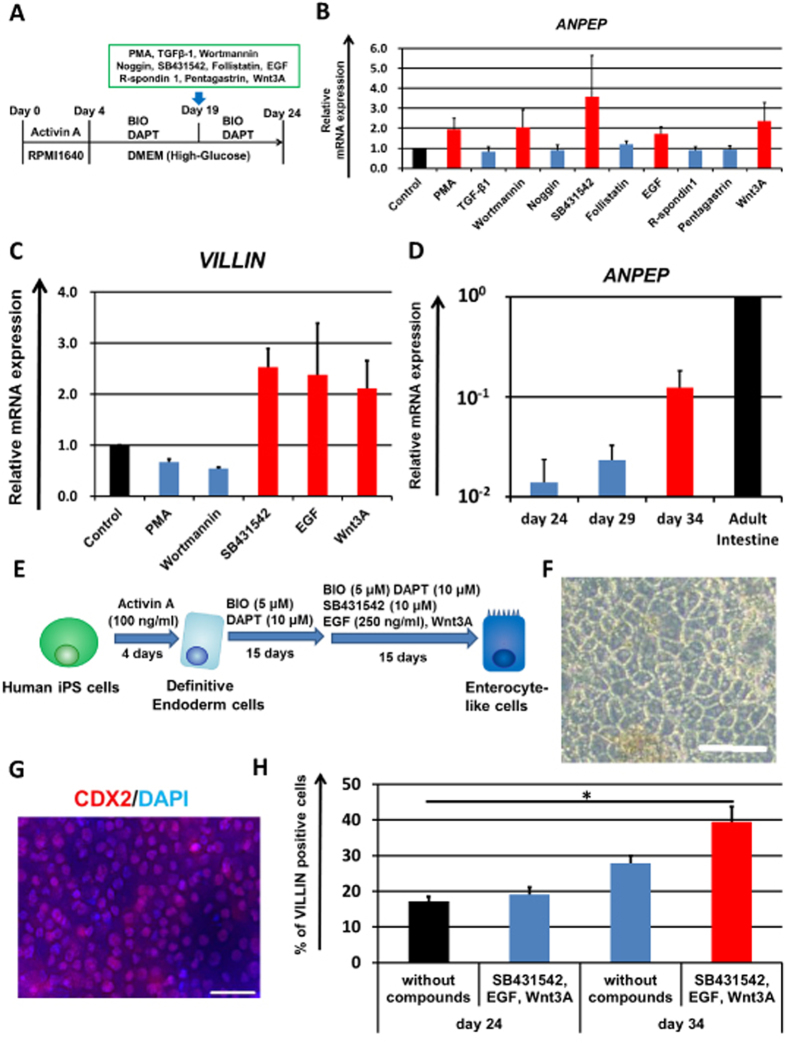
Promotion of enterocyte differentiation by combination treatment with three compounds and differentiation period extension. (**A**) The procedure for enterocyte differentiation from human iPS cells by treatment of compounds is presented. From day 19 to 24, the human iPS-derived intestinal cells were treated with the test compounds. (**B**) The gene expression levels of the enterocyte marker *ANPEP* in the test compound-treated human iPS-derived intestinal cells were measured by real-time RT-PCR analysis on day 24. On the y axis, the gene expression levels in “Control (untreated hiPS-ELCs)” were taken as 1.0. (**C**) On day 24, the gene expression levels of the enterocyte marker *VILLIN* in the PMA, Wortmannin, SB431542, EGF or Wnt3A-treated human iPS-derived intestinal cells were measured by real-time RT-PCR analysis. On the y axis, the gene expression levels in “Control” were taken as 1.0. (**D**) Temporal gene expression levels of *ANPEP* in the human iPS cell-derived intestinal cells (day 24, 29, and 34) were measured by real-time RT-PCR analysis. On the y axis, the gene expression levels in Adult Intestine were taken as 1.0. (**E**) The modified enterocyte differentiation protocol is illustrated. (**F**) A morphological image of human iPS-derived enterocyte-like cells is represented. Scale bar represents 100 μm. (**G**) Human iPS cell-derived enterocyte-like cells were assayed for the expression of intestinal marker CDX2 (Red) by immunohistochemistry. Nuclei were stained with DAPI (Blue). Scale bar represents 40 μm. (**H**) Percentages of VILLIN-positive cells in the SB431542, EGF, and Wnt3A-treated enterocyte-like cells were analyzed by flow cytometry analysis on day 24 and 34. Data are represented as the means ± S.E. (*n* ≧ 3). Statistical analysis was performed using the unpaired two-tailed student’s *t*-test. *P < 0.05.

To promote the enterocyte differentiation, we examined whether the treatment of compounds could promote enterocyte differentiation. First, we measured the temporal gene expression levels of the enterocyte marker *VILLIN* in the human iPS-derived intestinal cells (Fig. S1C). From day 19 to 24, the *VILLIN* gene expression levels were dramatically increased, suggested that enterocyte differentiation seems to be started after day 19. Therefore, we searched for compounds that could further promote enterocyte differentiation during this period. As shown in Fig. 1A, human iPS-derived intestinal cells (day 19) were treated with ten compounds respectively. These compounds are known to regulate signals which play important roles in intestinal epithelium differentiation or intestinal stem cell homeostasis. The gene expression levels of enterocyte marker *ANPEP* in the compound-treated human iPS-derived intestinal cells were examined by real-time RT-PCR on day 24. The gene expression levels of *ANPEP* were increased in the PMA, Wortmannin, SB431542, EGF, or Wnt3A-treated cells (Fig. 1B). Because *ANPEP* is highly expressed not only in the small intestine but also in other organs such as the liver and pancreas^27^, the gene expression levels of the enterocyte-specific marker *VILLIN* were also measured in the PMA, Wortmannin, SB431542, EGF, or Wnt3A-treated cells (Fig. 1C). The *VILLIN* gene expression levels were also increased in the SB431542, EGF, or Wnt3A-treated cells. These results might suggest that SB431542, EGF and Wnt3A treatment could promote enterocyte-like cell differentiation. In addition, the gene expression level of enterocyte marker *LACTASE* was increased by simultaneous treatment with SB431542, EGF and Wnt3A as compared with individual treatment with any one of these compounds (Fig. S2). The gene expression levels of *ANPEP* were decreased by removing one of three compounds (SB431542, EGF, or Wnt3A) (Fig. S3A). These results suggest that all these compounds play an important role in enterocyte-like cell differentiation. However, it has been reported that Wnt3A removal^28^ or TGFβ treatment^29^ could promote enterocyte differentiation. These reports contradict our present findings, which suggested that EGF, Wnt3A, and SB431542 treatment might promote enterocyte-like cell differentiation. To investigate this contradiction, we first examined the role of Wnt3A in enterocyte-like cell differentiation. We found that the *LGR5* (an intestinal stem cell marker) expression level was maintained and enhanced during the enterocyte differentiation by Wnt3A treatment (from day 19 to 34, Fig. S3B). In addition, most of the VILLIN-positive hiPS-ELCs were highly proliferative (Fig. S3D, left). It is known that Wnt3A signal is essential for maintenance of intestinal stem cells and transiently amplifying cells^30^. Taken together, we considered that our hiPS-ELCs required Wnt3A treatment, because the hiPS-ELCs were not terminally differentiated, but still have characters as intestinal progenitor/stem cell-like cells. Next, we examined the role of SB431542 in our enterocyte-like cell differentiation. Interestingly, the cell viability was decreased by SB431542 removal (Fig. S3C). Because the generated enterocyte-like cells were a heterogeneous population, we next examined the cellular changes of VILLIN-positive hiPS-ELCs in response to SB431542 treatment. By SB431542 removal, the percentages of Ki67 (a cellular proliferation marker; Fig. S3D)-positive cells and Annexin V (an early apoptosis marker; Fig. S3E)-positive cells among VILLIN-positive cells were decreased and increased, respectively. These results suggest that the inhibition of TGFβ signals could maintain the proliferative activity of VILLIN-positive enterocyte-like cells, and could also rescue these cells from apoptosis. Consequently, the differentiation to VILLIN-positive enterocyte-like cells was promoted by SB431542 treatment. Consistent with this notion, it has been reported that incubation of Caco-2 cells with TGFβ resulted in a significant decrease in cell viability and increased cell apoptosis^31^. The molecular mechanisms underlying the promotion of differentiation by EGF, SB431542, and Wnt3A should be further examined in the future.

To further promote enterocyte differentiation, the enterocyte differentiation period was optimized. In Fig. 1A–C, the human iPS-derived intestinal cells were treated with SB431542, EGF and Wnt3A for 5 days, versus 10 or 15 days in Fig. 1D. The gene expression levels of *ANPEP* in the SB431542, EGF and Wnt3A-treated cells (day 24, 29, or 34) were analyzed by real-time RT-PCR analysis. The *ANPEP* expression levels on day 34 were higher than those on day 24 and 29, suggesting that longer period of SB431542, EGF and Wnt3A treatment facilitates the enterocyte differentiation. Taken together, these results showed that the enterocyte differentiation could be promoted by treatment with the three compounds and extension of the differentiation period (Fig. 1E). After the enterocyte differentiation, epithelial-like cells were observed (Fig. 1F).

To examine the efficiency of enterocyte differentiation, the percentage of cells positive for the intestinal marker caudal type homeobox 2 (CDX2) or the enterocyte marker VILLIN were measured (Fig. 1G,H, respectively). The hiPS-ELCs were almost homogeneously positive for CDX2. This result suggests that almost all of the undifferentiated human iPS cells were successfully differentiated into intestinal lineage cells. The percentage of VILLIN-positive cells in the SB431542, EGF and Wnt3A-treated cells (day 34) was approximately 40%, although that in non-treated cells (day 24) was approximately 17%. In addition, the percentage of MUCIN2-positive secretory goblet cells was also increased (from 0.9% to 2.4%) by extending the differentiation period and treating the cells with three compounds (Fig. S4D). These results suggest that the intestinal epithelium differentiation was promoted by treatment with the compounds and extension of the differentiation period.

### Expression analysis of transporters in the human iPS-derived enterocyte-like cells

**Figure 2:**
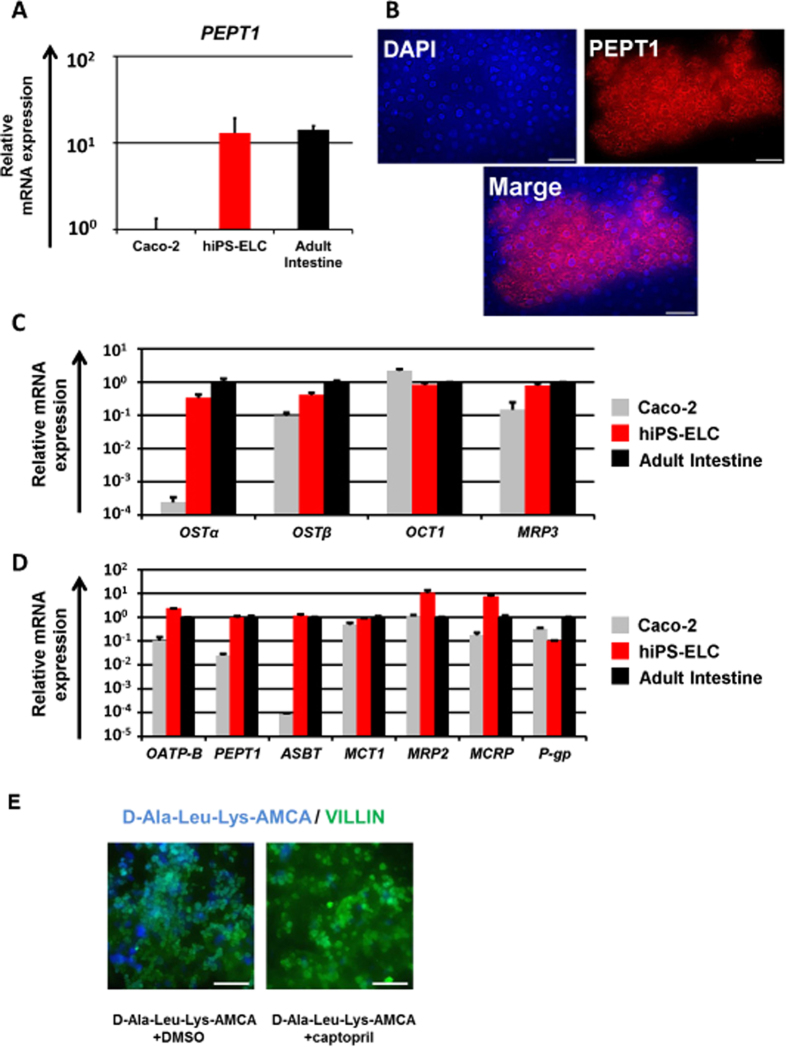
Expression analyses of intestinal transporters in the human iPS-derived enterocyte-like cells. Human iPS-derived enterocyte-like cells (hiPS-ELCs) were differentiated according to the protocol described in Fig. 1E. (**A**) The gene expression levels of *PEPT1* in Caco-2 cells, hiPS-ELCs, and Adult Intestine were measured by real-time RT-PCR analysis. On the y axis, the gene expression levels in Caco-2 cells were taken as 1.0. (**B**) The hiPS-ELCs were assayed for expression of PEPT1 (Red) by immunohistochemistry. Nuclei were stained with DAPI (Blue). Scale bars represent 40 μm. (**C,D**) The gene expression levels of apical transporters (**C**) and basolateral transporters (**D**) in the Caco-2 cells, hiPS-ELCs, and Adult Intestine were measured by real-time RT-PCR analysis. On the y axis, the gene expression levels in Adult Intestine were taken as 1.0. Data are represented as the means ± S.E. (*n* ≧ 3). Statistical analysis was performed using the unpaired two-tailed student’s *t*-test. *P < 0.05. (**E**) After the enterocyte differentiation, the hiPS-ELCs were treated with or without 100 μM captopril for 24 hr. The hiPS-ELCs were treated with D-Ala-Leu-Lys-AMCA (blue) for 4 hr. After the uptake of D-Ala-Leu-Lys-AMCA, the cells were fixed, and stained with anti-VILLIN antibodies (green). Scale bars represent 50 μm.

Various transporters, such as PEPT1 and P-gp, are expressed at high levels in the enterocytes. Although PEPT1 mediates the absorption of drugs with a peptide-like structure^32^, such as β-lactam antibiotics, the PEPT1 expression levels in Caco-2 cells are significantly lower than those in the human small intestine^25^. Here, the PEPT1 expression levels in the hiPS-ELCs were compared with those in Caco-2 cells and the human adult intestine. The *PEPT1* gene expression level in the hiPS-ELCs was approximately 13 times higher than that in Caco-2 cells, and was similar to that in the human adult intestine (Fig. 2A). Moreover, immunohistochemical analysis showed that the hiPS-ELCs were positive for PEPT1 (Fig. 2B). These results suggest that the PEPT1-mediated drug absorption might be more accurately evaluated by using the hiPS-ELCs than Caco-2 cells. The gene expression levels of other transporters expressed in the enterocytes^2^ were also measured by real-time RT-PCR analysis. The gene expression levels of transporters that are expressed on the apical surface of enterocytes were slightly lower in the hiPS-ELCs than in the human adult intestine, although this difference was not statistically significant (Fig. 2C). The gene expression levels of transporters that are expressed on the basolateral surface of enterocytes were similar between the hiPS-ELCs and the human adult intestine (Fig. 2D). However the gene expression levels of *P-gp* in the hiPS-ELCs were significantly lower than that in the human adult intestine. To examine the peptide transporting activities, the hiPS-ELCs were treated with fluorescent-labeled tripeptide (D-Ala-Leu-Lys-AMCA). The hiPS-ELCs could successfully uptake D-Ala-Leu-Lys-AMCA (Fig. 2E). In addition, this uptake was inhibited by PEPT1 inhibitor (Captopril) treatment, suggesting that uptake of D-Ala-Leu-Lys-AMCA in the hiPS-ELCs depends on PEPT1 activity. Taken together, these findings indicated that drug intestinal absorption through transporters including PEPT1 may be accurately predicted by using the hiPS-ELC model.

### Evaluation of CYP3A4 expression and activity level, and its induction potency in the human iPS-derived enterocyte-like cells

**Figure 3:**
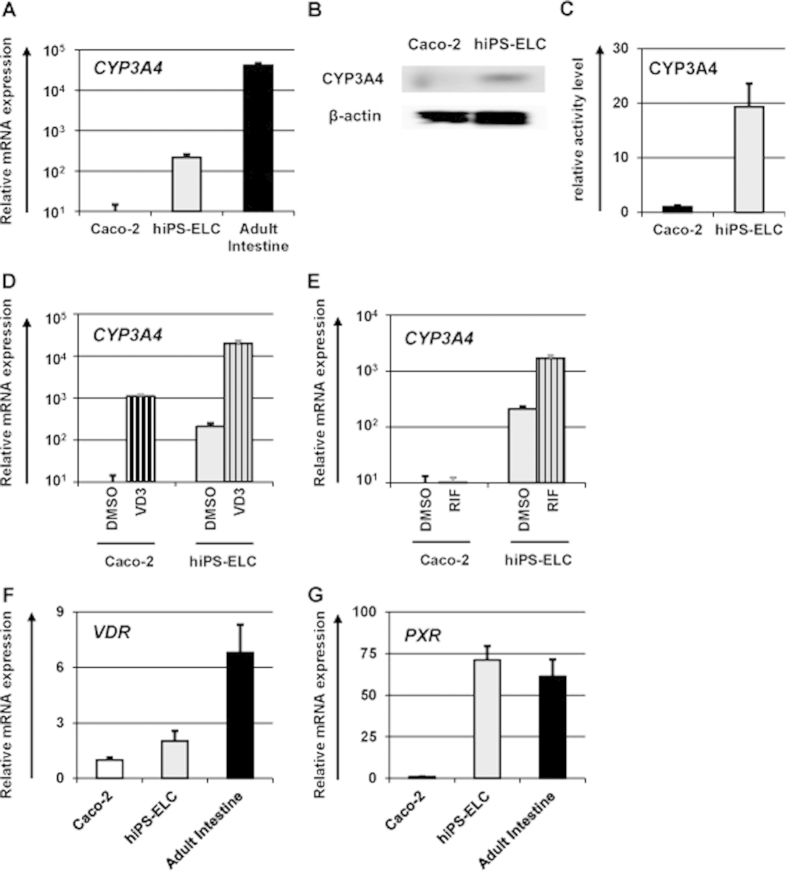
CYP3A4 expression level and induction potency in the human iPS-derived enterocyte-like cells. Human iPS cell-derived enterocyte-like cells (hiPS-ELCs) were differentiated according to the protocol described in Fig. 1E. (**A**) The gene expression levels of *CYP3A4* in Caco-2 cells, hiPS-ELCs, and Adult Intestine were measured by real-time RT-PCR analysis. On the y axis, the gene expression level in Caco-2 cells was taken as 1.0. (**B**) The CYP3A4 protein expression levels in the Caco-2 cells and hiPS-ELCs were measured by western blotting analysis. (**C**) The CYP3A4 activity levels in the Caco-2 cells and hiPS-ELCs were measured by CYP3A4-Glo assay kit. (**D,E**) The CYP3A4 induction potency was examined in the hiPS-ELCs and Caco-2 cells. The hiPS-ELCs and Caco-2 cells were treated with 100 nM 1,25-dihidroxyvitamin D3 (VD3) (**D**) or 20 μM rifampicin (RIF) (**E**) for 24 hr or 48 hr, respectively, and then the gene expression levels of *CYP3A4* were measured by real-time RT-PCR analysis. On the y axis, the gene expression levels of *CYP3A4* in Caco-2 cells treated with DMSO (Solvent) were taken as 1.0. (**F,G**) The gene expression levels of VDR (**F**) and PXR (**G**) in the hiPS-ELCs and Caco-2 cells were examined by real-time RT-PCR analysis. On the y axis, the gene expression levels of VDR and PXR in Caco-2 cells were taken as 1.0. The data are represented as the means ± S.E. (*n* ≧ 3).

It is known that CYP3A4 is a dominant drug metabolism enzyme in the enterocytes^33^. Although CYP3A4-mediated intestinal metabolism is a significant factor in oral drug bioavailability, the CYP3A4 expression levels in Caco-2 cells are significantly lower than those in the human small intestine^25^. Here, the CYP3A4 gene and protein expression levels were compared between the hiPS-ELCs and Caco-2 cells by real-time RT-PCR and western blotting analysis (Fig. 3A,B, respectively). The results showed that *CYP3A4* expression levels in the hiPS-ELCs were approximately 16 times higher than those in Caco-2 cells. Consistently, CYP3A4 protein expression levels in the hiPS-ELCs were higher than those in Caco-2 cells. In addition, the CYP3A4 activity levels in the hiPS-ELCs were approximately 20 times higher than those in Caco-2 cells (Fig. 3C). However, the *CYP3A4* expression levels in the hiPS-ELCs were approximately 100 times lower than those in the human adult intestine.

To examine the CYP3A4 and P-gp induction potencies in the hiPS-ELCs, the hiPS-ELCs were treated with the CYP3A4 inducers. It is known that 1,25-dihidroxyvitamin D3^12^,^34^ treatment induces CYP3A4 expression in Caco-2 cells, although rifampicin treatment does not induce CYP3A4 expression in Caco-2 cells^12^. This difference was attributed to the low expression of pregnane X receptor (PXR), which is known to be a nuclear receptor of rifampicin, in Caco-2 cells^35^. In Fig. 3D,E, the hiPS-ELCs and Caco-2 cells were treated with 1,25-dihidroxyvitamin D3 or rifampicin, and then the gene expression levels of *CYP3A4* were examined by real-time RT-PCR analysis. The *CYP3A4* expression levels in the 1,25-dihidroxyvitamin D3- or rifampicin-treated hiPS-ELCs were significantly increased as compared with those in the DMSO-treated hiPS-ELCs (Fig. 3D,E, respectively). On the other hand, the *CYP3A4* expression levels in Caco-2 cells were increased by 1,25-dihidroxyvitamin D3 treatment, but not by rifampicin treatment. In addition, the gene expression of *P-gp* in the hiPS-ELCs could also be induced by 1,25-dihidroxyvitamin D3 or rifampicin treatment (Fig. S5). We confirmed that the vitamin D receptor (*VDR*) expression levels in hiPS-ECLs and Caco-2 cells were similar to each other (Fig. 3F), although the *PXR* expression levels in hiPS-ELCs were significantly higher than those in Caco-2 cells (Fig. 3G). This might have been the reason why the rifampicin-mediated CYP3A4 and P-gp inductions were successfully confirmed in the hiPS-ELCs, but not in Caco-2 cells. These results suggest that CYP3A4 and P-gp induction by the test drugs would be evaluable by using the hiPS-ELCs.

### Tight junction and barrier formation in the human iPS-derived enterocyte-like cell monolayers

**Figure 4:**
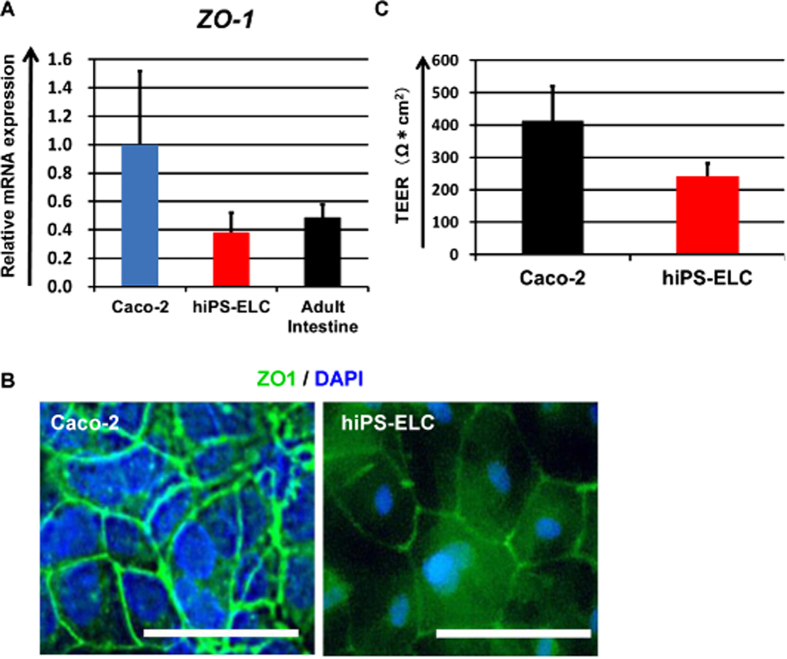
Analysis of barrier formation capacity in the human iPS-derived enterocyte-like cell monolayers. (**A**) The gene expression levels of *ZO-1* in Caco-2 cells, human iPS-derived enterocyte-like cells (hiPS-ELCs) and Adult Intestine were examined by real-time RT-PCR analysis. On the y axis, the gene expression levels in Caco-2 cells were taken as 1.0. (**B**) Immunostaining analysis of ZO-1 (Green) in the hiPS-ELCs and Caco-2 cells was performed. Nuclei were stained with DAPI (Blue). Scale bars represent 40 μm. (**C**) TEER values of Caco-2 cell monolayers and hiPS-ELC monolayers were measured by Millicell-ERS. All data are represented as the means ± S.E. (*n* ≧ 3).

To examine whether the hiPS-ELCs would be applicable to drug absorption studies, the expression levels of tight junction-related genes and barrier function in the hiPS-ELCs were evaluated. The gene and protein expression levels of ZO-1 in the hiPS-ELCs were measured by real-time RT-PCR and immunohistochemical analysis (Fig. 4A,B, respectively). The gene expression levels of *ZO-1* in the hiPS-ELCs were similar to those in the human adult intestine, and were lower than those in Caco-2 cells. In addition, both the hiPS-ELCs and Caco-2 cells were positive for ZO-1. Barrier function in the hiPS-ELC and Caco-2 cell monolayers was analyzed by TEER measurements and FD-4 permeability tests (Fig. 4C and S6, respectively). The TEER values in the hiPS-ELC and Caco-2 cell monolayers were approximately 240 and 400 Ω*cm^2^, respectively. Moreover, the hiPS-ELC monolayers had higher FD-4 permeability than Caco-2 cells, but lower than undifferentiated human iPS cells. These results suggest that the hiPS-ELC monolayers have weaker barrier function than the Caco-2 cell monolayers. Importantly, it is known that the TEER values in Caco-2 cell monolayers are higher than those in the small intestine^13^,^36^. Therefore, the hiPS-ELC monolayers might be a more suitable *in vitro* model for evaluating the absorption of hydrophilic drugs than the Caco-2 cell monolayers.

## Discussion

The main purpose of this study was to generate hiPS-ELCs that could be used for drug absorption and metabolism studies. The efficiency of enterocyte differentiation from human iPS cells was significantly improved by using EGF, SB431542, and Wnt3A treatment, and by the extension of differentiation period (Fig. 1). The hiPS-ELCs expressed CYP3A4 and PEPT1 at higher levels than Caco-2 cells (Figs 2 and 3, respectively). In addition, the hiPS-ELCs showed CYP3A4 and P-gp induction potency (Fig. 3 and S3, respectively) and barrier forming potency (Fig. 4).

We identified that treatment with the combination of EGF, SB431542 (TGFβ signal inhibitor) and Wnt3A treatments could enhance the enterocyte differentiation efficiency (Fig. 1). At the end of differentiation (day 34), the *LGR5* expression was maintained at a high level (Fig. S3B), and approximately 80% of VILLIN-positive hiPS-ELCs were proliferative (Fig. S3D). Because it is known that EGF and Wnt3A are essential for maintenance of the self-renewing intestinal organoids^28^,^37^,^38^, EGF and Wnt3A treatments might support the proliferation of the human iPS-derived intestinal stem/progenitor cells. Therefore, EGF and Wnt3A treatment might have increased the enterocyte differentiation efficiency by enhancing the proliferation of human iPS-derived intestinal stem/progenitor cells. In addition, the removal of SB431542 resulted in a significant decrease of proliferating VILLIN-positive cells, and an increase of apoptotic VILLIN-positive cells (Figs S3D and S3E, respectively). Thus the inhibition of TGFβ signals might also have contributed to the survival and proliferation of human iPS-derived intestinal stem/progenitor cells.

Although the percentage of VILLIN-positive hiPS-ELCs was significantly improved by using EGF, SB431542, and Wnt3A treatment, and differentiation period extension (Fig. 1), the percentage of VILLIN-negative cells (i.e. non-enterocytes) was still more than 50%. Because the percentage of CDX2-positive cells was almost 100% (Fig. 1G), the VILLIN-negative cells seemed to be immature cells in intestinal lineage, such as hindgut cells. The higher purity of enterocyte populations is, the more precise drug absorption and metabolism experiments would be performed. Therefore, we consider that further enhancement of the enterocyte differentiation efficiency is required. Because we have previously reported that hepatocyte differentiation from human pluripotent stem cells could be promoted by gene transfer technology^39^,^40^ and 3-dimensional cell culture system^41^, these technologies might also be useful for improvement of the enterocyte differentiation method.

In Fig. 2, the gene expression levels of *PEPT1* in the hiPS-ELCs were approximately 13 times higher than those in Caco-2 cells. It is known that the PEPT1 expression levels in the human duodenum and ileum are approximately 54 and 88 times higher than that in the human large intestine^42^. As shown in Fig. 4, the TEER value in the hiPS-ELC monolayers was lower than that in Caco-2 cell (human colon carcinoma cell line) monolayers. It has been reported the TEER value in the small intestine is lower than that in the colon^36^. Taken together, these finding indicate that the hiPS-ELC model might faithfully reproduce the human small intestine rather than the human large intestine. In addition, the hiPS-ELCs expressed CYP3A4, while Caco-2 cells express CYP3A4 at a low level. Because CYP3A4-mediated intestinal metabolism is a significant factor in oral drug bioavailability, the hiPS-ELCs have potential for use in the accurate prediction of oral drug bioavailability. Moreover, the hiPS-ELCs have CYP3A4 and P-gp induction potency, and thus the hiPS-ELC model might be useful for screening drug candidates which have risk to induce CYP3A4 and P-gp expression levels.

In conclusion, we have succeeded in generating the hiPS-ELCs which have a potential to be utilized in both drug absorption and metabolism studies. In addition, the hiPS-ELC models, which can be evaluate CYP3A4 and P-gp induction potency by test drugs, were successfully generated. We believe that our hiPS-ELC model will greatly accelerate the safe and efficient discovery and development of novel drugs.

## Additional Information

**How to cite this article**: Ozawa, T. *et al.* Generation of enterocyte-like cells from human induced pluripotent stem cells for drug absorption and metabolism studies in human small intestine. *Sci. Rep.* **5**, 16479; doi: 10.1038/srep16479 (2015).

## Supplementary Material

Supplementary Information
