# Supplementary material for: Generation of enterocyte-like cells from human induced pluripotent stem cells for drug absorption and metabolism studies in human small intestine
Source: Sci Rep. 2015 Nov 12;5:16479. doi: 10.1038/srep16479 (PMC4642303; doi:10.1038/srep16479)
Supplement: Supplementary Information [file srep16479-s1.pdf]

Supplemental Information

Generation of enterocyte-like cells from human induced pluripotent stem cells for drug absorption and metabolism studies in human small intestine

Tatsuya Ozawa[1,2#], Kazuo Takayama[1,2,3#], Ryota Okamoto[1,2], Ryosuke Negoro[1], Fuminori Sakurai[1,4], Masashi Tachibana[1], Kenji Kawabata[5,6], Hiroyuki Mizuguchi[1,2,3,7*]

[1]Laboratory of Biochemistry and Molecular Biology, Graduate School of Pharmaceutical Sciences, Osaka University, Osaka 565-0871, Japan; [2]Laboratory of Hepatocyte Differentiation, National Institute of Biomedical Innovation, Health and Nutrition, Osaka 567-0085, Japan; [3]iPS Cell-based Research Project on Hepatic Toxicity and Metabolism, Graduate School of Pharmaceutical Sciences, Osaka University, Osaka 565-0871, Japan; [4]Laboratory of Regulatory Sciences for Oligonucleotide Therapeutics, Clinical Drug Development Project, Graduate School of Pharmaceutical Sciences, Osaka University Osaka 565-0871, Japan; [5]Laboratory of Stem Cell Regulation, National Institute of Biomedical Innovation, Health and Nutrition, Osaka 567-0085, Japan; [6]Laboratory of Biomedical Innovation, Graduate School of Pharmaceutical Sciences, Osaka University, Osaka 565-0871, Japan; [7]Global Center for Medical Engineering and Informatics, Osaka University, Osaka 565-0871, Japan.

# These authors contributed equally to this work.

*To whom correspondence should be addressed: Dr. Hiroyuki Mizuguchi
Laboratory of Biochemistry and Molecular Biology, Graduate School of Pharmaceutical Sciences, Osaka University, 1-6 Yamadaoka, Suita, Osaka 565-0871, Japan.
Phone: +81-6-6879-8185
FAX: +81-6-6879-8186
E-mail: mizuguch@phs.osaka-u.ac.jp

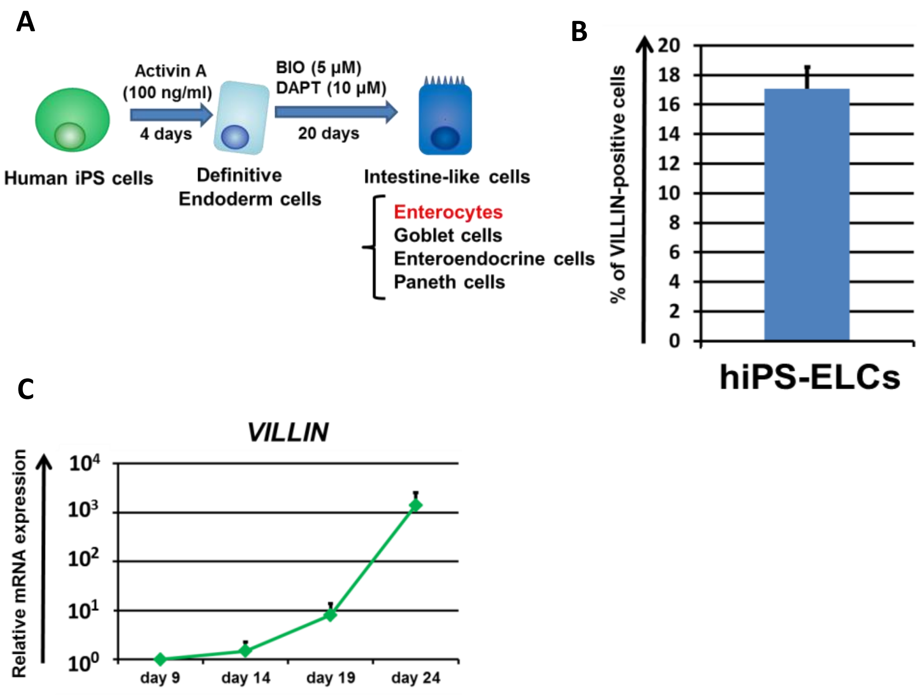

Supplemental Figure 1 The expression analysis of VILLIN in the intestinal differentiation from human iPS cells

(A) An overview of intestinal differentiation from human iPS cells is presented. Under the feeder-free condition, human iPS cells were differentiated into the definitive endoderm cells and intestinal cells by using Activin A, BIO, and DAPT. (B) On day 24, the percentage of enterocyte maker VILLIN-positive cells in the hiPS-ELCs was analyzed by flow cytometry analysis. (C) The temporal gene expression levels of the enterocyte marker *VILLIN* in the human iPS-derived intestinal cells (day 9, 14, 19, and 24) were examined by real-time RT-PCR analysis. On the y axis, the gene expression levels on “day 9” were taken as 1.0.

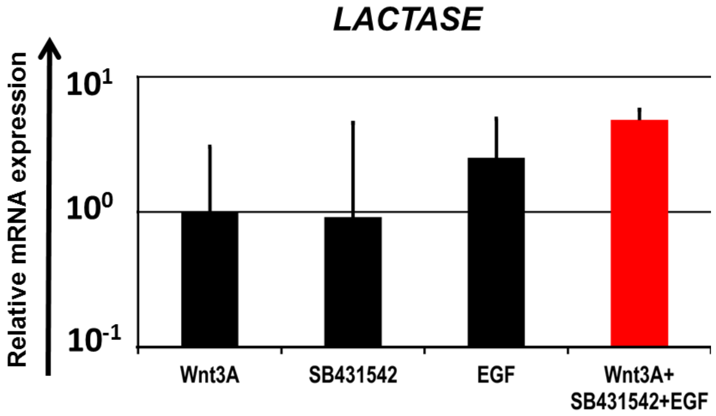

Supplemental Figure 2 The gene expression analysis of the enterocyte marker *LACTASE* in the Wnt3A, SB431542, and EGF-treated human iPS-derived intestinal cells

From day 19 to 24, human iPS-derived intestinal cells were treated with Wnt3A, SB431542, EGF, or Wnt3A + SB431542 + EGF. The gene expression levels of the enterocyte marker *LACTASE* were measured by real-time RT-PCR analysis on day 24. On the y axis, the gene expression levels in Wnt3A-treated cells were taken as 1.0.

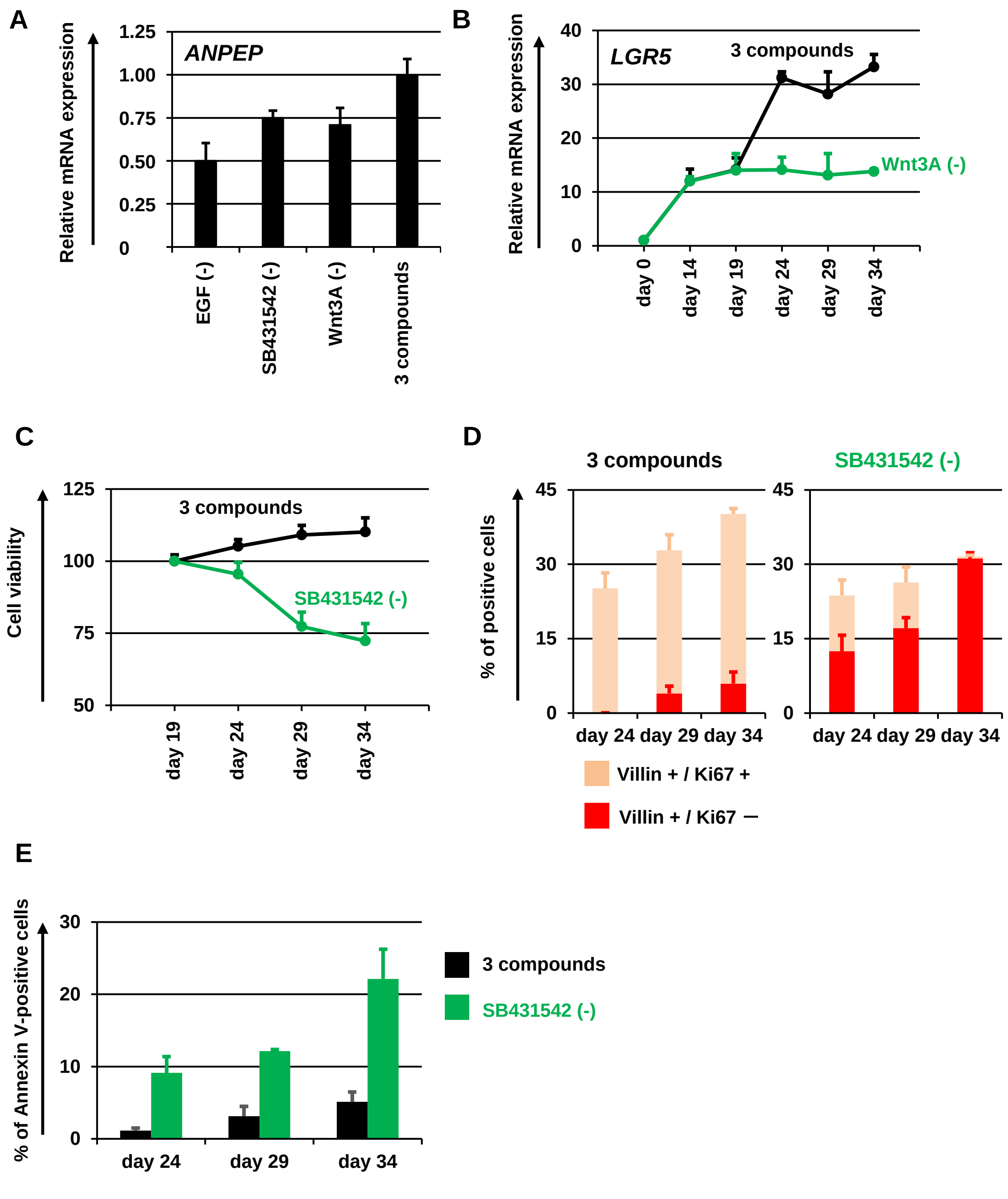

Supplemental Figure 3 The functions of Wnt3A and SB431542 in the hiPS-ELCs

(A) From day 19 to 34, human iPS-derived intestinal cells were treated with the compounds Wnt3A, SB431542 and EGF, or with 2 of these 3 compounds. The gene expression levels of the enterocyte marker *ANPEP* were measured by real-time RT-PCR analysis on day 34. On the y axis, the gene expression levels in the cells treated with all 3 compounds were taken as 1.0. (B) From day 19 to 34, human iPS-derived intestinal

cells were treated with Wnt3A, SB431542 and EGF, or with only SB431542 and EGF. The temporal gene expression levels of intestinal stem cell marker *LGR5* were measured by real-time RT-PCR analysis. On the y axis, the gene expression levels in undifferentiated human iPS cells (day 0) were taken as 1.0. (C-E) From day 19 to 34, human iPS-derived intestinal cells were treated with the compounds Wnt3A, SB431542 and EGF, or with only Wnt3A and EGF. The temporal cell viability was examined by WST-8 assay (C). On the y axis, the cell viability on day 19 was taken as 100. The temporal percentage of VILLIN- and Ki67-positive cells was examined by FACS analysis (D). The temporal percentage of Annexin V-positive cells in the hiPS-ELCs (VILLIN-positive cells) was also examined by FACS analysis.

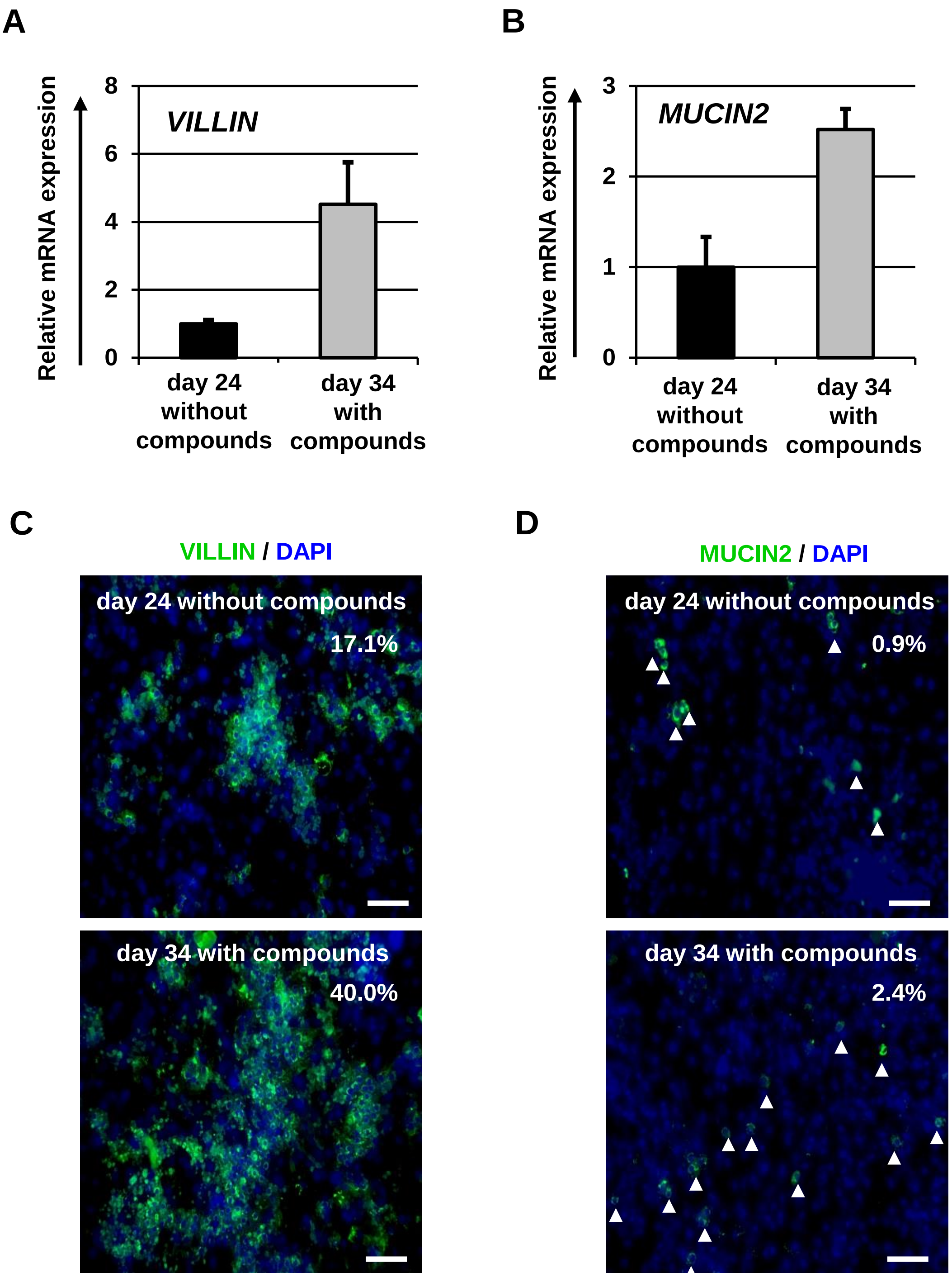

Supplemental Figure 4 Expression analysis of VILLIN and MUCIN2 in the hiPS-ELCs

(A-D) The expression levels of VILLIN (enterocyte marker; A and C)- or MUCIN2 (secretory goblet cell marker; B and D)-positive cells in the non-treated hiPS-ELCs (day 24; day 24 without compounds) and in the SB431542, EGF, and Wnt3A-treated hiPS-ELCs (day 34; day 34 with compounds) were analyzed by

real-time RT-PCR (A and B) and semi-quantitative immunohistochemical analysis (C and D). Scale bars represent 50 μm. MUCIN2-positive cells are indicated by white arrowhead.

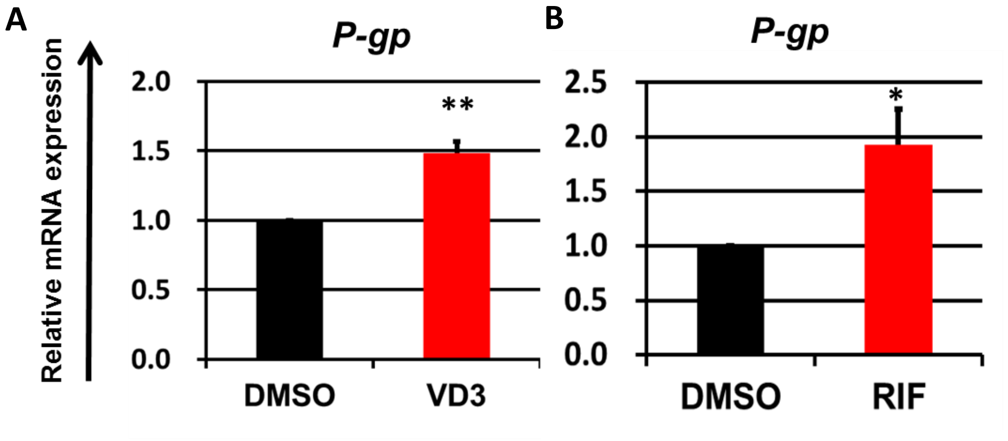

Supplemental Figure 5 P-gp induction potency in the human iPS-derived enterocyte-like cells

(A, B) The induction potency of P-gp was examined in the human iPS-derived enterocyte-like cells (hiPS-ELCs). The hiPS-ELCs were treated with 100 nM 1,25-dihidroxyvitamin D3 (VD3) (A) or 20 μM rifampicin (RIF) (B) for 24 hr or 48 hr, respectively, and then the gene expression levels of *P-gp* were measured by real-time RT-PCR analysis. On the y axis, the gene expression levels of *P-gp* in the hiPS-ELCs treated with DMSO (Solvent) were taken as 1.0. All data are represented as the means ±S.E. ($n \geqq 3$) * p<0.05; ** p<0.01.

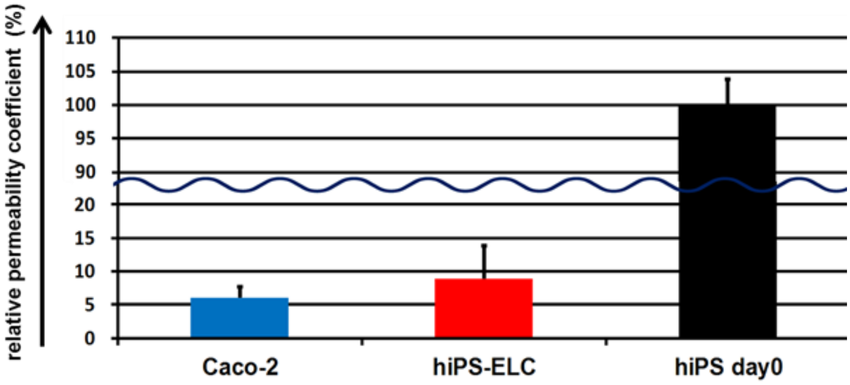

Supplemental Figure 6 FD-4 permeability activity in the human iPS cell-derived enterocyte-like cells, Caco-2 cells, and undifferentiated human iPS cells

The permeability activity in Caco-2 cells, human iPS-derived enterocyte-like cells (hiPS-ELCs), and undifferentiated human iPS cells (hiPS day 0) were examined by treating FD-4. On the y axis, the permeability activity in undifferentiated human iPS cells was taken as 100%.

Antigen	Type	Company	Dilution
CDX2	mouse	Abcam (ab15258)	1:100
PEPT1	rabbit	Santa Cruz (sc-20653)	1:100
ZO-1	rabbit	Santa Cruz (sc-10804)	1:100
VILLIN	rabbit	Abcam (ab109516)	1:100
MUCIN2	rabbit	Santa Cruz (sc-15334)	1:100

Antigen	Company	Dilution
Anti-mouse IgG Antibody	Invitrogen (A11032)	1:1000
Anti-Rabbit IgG Antibody	Invitrogen (A11012)	1:1000
Anti-Rabbit IgG Antibody	Invitrogen (A21206)	1:1000

Table S1 List of antibodies used for immunohistochemistry

Gene symbol	Sequence (forward/reverse ; 5' to 3')
GAPDH (glyceraldehyde 3-phosphate dehydrogenase)	GGGAACAACATACAGTGACGC/CCCCACTCTGAAAATGAGGA
ANPEP (aminopeptidase N)	AAGCCTGTTTCCTCGTTGTC /AACCTCATCCAGGCAGTGAC
VILLIN	CTGCAGCCAGCTTGCCACAACT/CACGCCCCCTTTCCGGATCAC
CYP3A4 (cytochrome P 450 3A4)	AGATGCCTTTAGGTCCCAATGGG/GCTGGAGATAGCAATGTTCGT
PEPT1 (peptide transporter 1)	TGCTCAGGGGATAACCAAAG/ACCTGCCAGGAGCACGTC
OATP-B (organic anion-transporting polypeptide B)	TGATTGGCTATGGGGCTATC/CATATCCTCAGGGCTGGTGT
ASBT (apical sodium-dependent bile acid transporter)	TATAGGATGCTGCCCTGGAG/AGTGTGGAGCATGTGGTCAT
MCT1 (monocarboxylate transporter 1)	GCGATCCGCGCATATAAC/AACTGGACCTCCAACTGCTG
MRP2 (multidrug resistance associated protein 2)	TGAGCAAGTTTGAAACGCACAT/AGCTTCTCCTGCCGTCTCT
BCRP (breast cancer resistance protein)	TGCAACATGTACTGGCGAAGA/TCTTCCACAGCCCCAGG
P-gp (P-glycoprotein)	GCCAAAGCCAAAATATCAGC/TTCCAATGTGTTCGGCATTA
OCT1 (organic cation transporter 1)	TAATGGACCACATCGCTCAA/AGCCCCTGATAGAGCACAGA
OSTα (organic solute transporter α)	GAAGACCAATTACGGCATCC/AGTGAGGGCAAGTTCCACAG
OSTβ (organic solute transporter β)	GAGCTGCTGGAAGAGATGCT/TGCTTATAATGACCACCACAGC
MRP3 (multidrug resistance associated protein 3)	GTCCGCAGAATGGACTTGAT/TCACCACTTGGGGATCATTT
VDR (vitamin D receptor)	GCAGGTAAGTGCAGCCCCAG/GAACAGCTTGTCCACCCG
PXR (pregnane X receptor)	TCCGGAAAGATCTGTGCTCT/AGGGAGATCTGGTCCTCGA
ZO-1 (zonula occludens-1)	GGGAACAACATACAGTGACGC/CCCCACTCTGAAAATGAGGA
MUCIN2	GAGGGCAGAACCCGAAACC/GGCGAAGTTGTAGTCGCAGAG
LGR5 (leucine-rich repeat containing G protein-coupled receptor 5)	CTCCCAGGTCTGGTGTGTTG/GAGGTCTAGGTAGGAGGTGAAG

Table S2 List of primers used for real-time RT-PCR analysis
